# A mismatch in enzyme-redox partnerships underlies divergent cytochrome P450 activities between human hepatocytes and microsomes

**DOI:** 10.1038/s42003-025-08903-1

**Published:** 2025-11-06

**Authors:** Tashinga E. Bapiro, Scott Martin, Thomas S. Blacker, Stephen D. Wilkinson, Alexandra L. Orton, Niresh Hariparsad, Rhys D. O. Jones, Michael R. Duchen, Stephanie Harlfinger

**Affiliations:** 1 Drug Metabolism and Pharmacokinetics, Oncology Research and Development, AstraZeneca, Cambridge, UK; 2 Research Department of Cell & Developmental Biology, University College London, London, UK; 3 Drug Metabolism and Pharmacokinetics, Oncology Research and Development, AstraZeneca, 35 Gatehouse Park Drive, Boston, MA USA; 4 Present Address: DMPK, UCB Pharma, Berkshire, UK; 5 Present Address: NCE DMPK, Merck Healthcare KGaA, Darmstadt, Germany

**Keywords:** Oxidoreductases, Enzyme mechanisms, Pharmacokinetics

## Abstract

NADPH-P450 oxidoreductase (POR) is the accepted redox-partner for drug-metabolising cytochrome P450s (CYPs) over NADH-cytochrome *b*_*5*_ reductase (Cyt*b*_*5*_R). Accordingly, POR-centric NADPH-supplemented human liver microsomes (HLM) and recombinant CYP-POR systems complement human hepatocytes (HH) as drug-metabolism models. However, HH may exhibit inexplicably lower CYP activities relative to NADPH-HLM, particularly for CYP3A4-substrates. Here, we show the phenomenon can manifest as disparate CYP phenotyping in which NADPH-HLM and recombinant CYP-POR systems incorrectly identify CYP3A4 while HH accurately assign the main-metabolising CYP exemplified by CYP1A2 for savolitinib. Mechanistically, we serendipitously discover that HH CYP3A4-mediated midazolam-metabolism is increased by gefitinib and find that this can only be recapitulated in non-canonical Cyt*b*_*5*_R-dependent NADH-HLM and recombinant CYP3A4-Cyt*b*_*5*_R. We conclude that Cyt*b*_*5*_R is important for HH-CYP3A4 and show HH-consistent CYP3A4-activities in NADH-HLM. Imaging NADH/NADPH in hepatocytes shows equivalent concentrations suggesting CYP redox-partnerships are cofactor-independent and likely influenced by protein-protein interactions as mimicking the dense-intracellular protein using albumin recapitulates HH savolitinib-metabolism in HLM.

## Abstract (web summary)

Distinct cytochrome P450 (CYP) activities associated with specific CYP-redox partnerships show that divergent enzyme activities between human hepatocytes and microsomes may be driven by a mismatch in redox-partner interactions.

## Introduction

Cytochrome P450 (CYP) is a superfamily of enzymes primarily localised to the liver and notable for catalysing an extensive set of reactions including bio-transformations that render small molecule therapeutics inactive^1–4^. As a result, FDA-approved in vitro assays that use human hepatocytes (HH) and human liver microsomes (HLM) to predict in vivo CYP-drug metabolism are an integral part of small molecule drug discovery programs^5,6^. However, HH have been shown to exhibit disproportionately lower CYP activities relative to NADPH-supplemented HLM (NADPH-HLM) as measured by substrate turnover, particularly for substrates of the most important CYP, CYP3A4^7–9^ that, in our experience at AstraZeneca, create uncertainty in drug discovery projects due to the absence of a satisfactory mechanistic explanation.

HH and HLM are recognised as mutually complementary workhorses of in vitro CYP studies with well-documented system-specific pros and cons^10–12^. The agreement of results from both models builds confidence in in vivo CYP-metabolism projections thus providing a sound rationale for iterative chemical refinements if needed, that are aimed primarily at the development of candidate drugs with reduced CYP-metabolic liabilities^5^. A key parameter associated with metabolic liability assessments is clearance (CL), and its derivation from in vitro intrinsic clearance (CL_int_ or V_max_/K_m_) has been described^13,14^. Also well-documented are scaling factors that normalise and scale-up to body weight giving an unbound scaled intrinsic clearance (CL_int,u_) that should be independent of the model^13–15^. Both models should, therefore, produce similar scaled CL_int,u_ values for predominantly CYP-cleared compounds. However, a number of compounds, the majority of which are CYP3A4 substrates^8^, display higher scaled CL_int,u_ values in NADPH-HLM relative to HH that are not understood and, of the possible explanations suggested over the years, none has proved satisfactory^7,9,16–20^. Recently, we reported higher intrinsic CYP activities for a set of CYP3A4 substrates in NADPH-HLM relative to HH as an explanation^21^ but could not identify the underlying cause. The absence of a comprehensive mechanistic explanation after many years of intense research effort could indicate fundamental gaps in our understanding of CYPs. With this perspective, the intricate CYP-redox partner interaction-dynamics stand out due to the lack of structural data as highlighted by Guengerich et al.^3^.

CYPs involved in drug metabolism are localised to endoplasmic reticulum (ER) membranes^22–24^ in close association with redox partners. NADPH P450 oxidoreductase (POR), thought to be the main CYP-redox partner, can transfer the required two electrons to CYPs^23–25^, however, the mechanistic basis underlying possible involvement in electron transfer, of another ER-associated protein, cytochrome *b*_*5*_, is not clear^26–28^. Regarding the cofactor, in vitro studies show that POR has a higher affinity for NADPH relative to NADH^29–31^. A second redox partner also localised to the ER, NADH cytochrome *b*_*5*_ reductase (Cyt*b*_*5*_R), cannot transfer electrons directly to CYP but can only do so via cytochrome *b*_*5*_ and prefers NADH as cofactor^32^. Much lower in vitro CYP-activities observed with Cyt*b*_*5*_R relative to POR^26,33^ support the general belief that Cyt*b*_*5*_R may not contribute significantly to drug metabolism. As a result, commercial recombinant CYP expression systems, which complement HH and HLM, co-express: the CYP of interest, POR and cytochrome *b*_*5*_, but not Cyt*b*_*5*_R (https://bioivt.com/subcellular-fractions/recombinant-cytochrome-p450-enzymes). Differences in affinity for cofactors mean POR and Cyt*b*_*5*_R activities in HLM can be separated based on the added cofactor^34^. Hence, supplementation of HLM with NADPH supports CYP-POR redox partnerships. However, similar affinities for their cognate cofactors coupled with much higher values for the opposite cofactor (K_m_ values: Cyt*b*_*5*_R for NADH, 6 µM; for NADPH, 1 mM and POR for NADPH, 2–15 µM; for NADH, 48 mM)^29–32^ raise important questions regarding mechanisms governing intracellular CYP redox-partner selection and whether these are recapitulated in NADPH-supplemented HLM incubations.

Key evidence supporting POR as the main electron provider to CYPs in vivo came from POR-knockdown studies in cell and in vivo models^35,36^. However, the associated off-target effects from POR-knockdown including hyperlipidaemia, reduction in bile acids and liver damage^36,37^, that would, on their own, impact CYP activities, make discernment of the impact of POR-knockdown per se, difficult. Interestingly, another in vivo study, devoid of potential CYP activity-altering off-target effects, demonstrated a major role for cytochrome *b*_*5*_ in the CYP-metabolism of specific substrates including midazolam^38^_._ However, the lack of clarity regarding the precise contribution or lack thereof, of cytochrome *b*_*5*_’s cognate redox partner, Cyt*b*_*5*_R^39^, critically, raises the question whether POR-centric HLM incubations accurately capture HH Cyt*b*_*5*_R-cytochome *b*_*5*_ activities and any associated effects on CYP-metabolism rates and routes.

Here, we show that the disproportionately lower HH CYP3A4 activities relative to NADPH-HLM can manifest as disparate CYP phenotyping in which NADPH-HLM and recombinant CYP-POR systems incorrectly identify CYP3A4 while HH accurately assign the main-metabolising CYP exemplified by CYP1A2 for savolitinib, a c-MET inhibitor^40^. Mechanistically, we serendipitously discovered that HH CYP3A4-mediated midazolam clearance is increased by gefitinib and find that this could only be recapitulated in non-canonical Cyt*b*_*5*_R-dependent NADH-HLM and recombinant CYP3A4-Cyt*b*_*5*_R. We conclude that Cyt*b*_*5*_R is important for HH-CYP3A4 and show HH-consistent CYP3A4-activities in NADH-HLM for multiple compounds, but the CYP1A2-activity is insufficient to capture HH savolitinib-metabolism. Imaging NADH/NADPH in hepatocytes showed equivalent concentrations of both cofactors suggesting that intracellular factors render CYP redox-partner selection cofactor-independent. We identified protein as a possible factor and faithfully recapitulated HH savolitinib-metabolism in NADH/NADPH-HLM by mimicking the dense-intracellular protein missing from HLM using albumin. Together, our work supports a CYP-dependent redox partner selection, underpinned by intracellular dynamics, as a key determinant of CYP metabolism rates and routes, which may explain the observed disconnect in scaled CL_int,u_ values determined in HH and NADPH-HLM for some CYP3A4 substrates.

## Results

### Non-CYP metabolism may mask lower HH CYP3A4 activities relative to NADPH-HLM

**Fig. 1:**
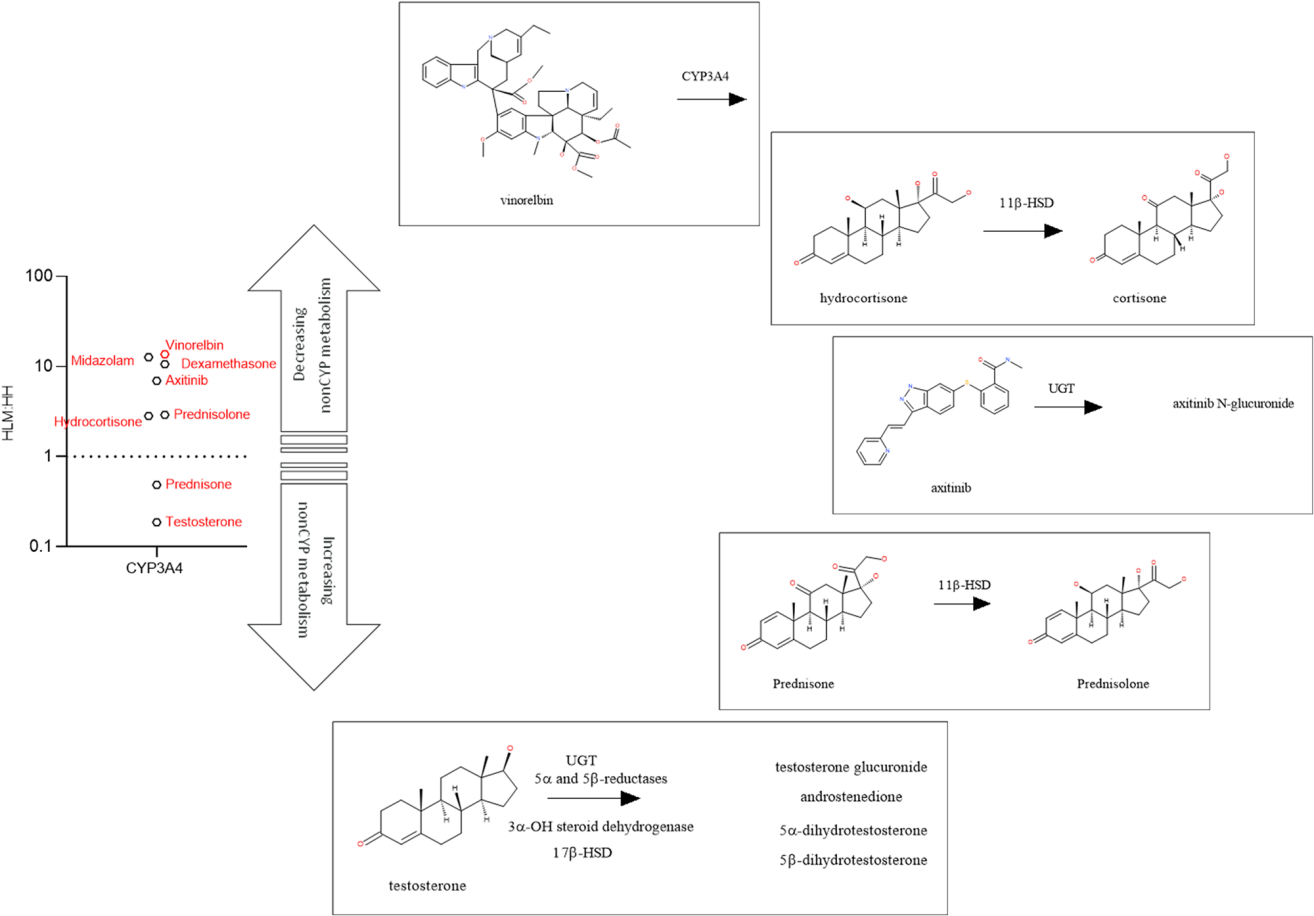
Non-CYP metabolism in HH may mask lower HH CYP3A4 activities relative to NADPH-HLM. Ratio of HLM:HH scaled CL_int,u_ values for eight compounds considered to be CYP3A4 substrates. Each point represents data from three independent determinations in HH and NADPH-HLM. The routes of metabolism in vitro and in vivo have been published^42–51^.

Data generated from our group and others, show that most compounds exhibiting lower metabolism rates in HH relative to NADPH-HLM are CYP3A4 substrates^8,41^. An important question which arises is why some compounds show apparent concordance or higher metabolism rates in HH. Previous studies examined disappearance of substrate only, whereas, we have extended this to understand the major metabolites produced in NADPH-HLM and HH. A detailed look at eight CYP3A4 substrates (Fig. 1) revealed that although NADPH-HLM showed CYP3A4 as the dominant metabolising-CYP, HH and in vivo data indicates that non-CYP routes not supported in HLM are also present for some of the compounds^42–51^. Indeed, we have shown that for a set of compounds from our internal 5-azaquinazoline series, the disproportionately low HH-CYP3A4 activities relative to NADPH-HLM are masked by the cytosolic enzyme, aldehyde oxidase^21^. Testosterone is a well-known CYP3A4 substrate based on in vitro data in NADPH-HLM and recombinant CYP-POR systems^42^. However, it is also well known that a major route of testosterone metabolism in the liver is via glucuronidation and reduction by 5α and 5β-reductases^44^. The low rate of HH CYP3A4-mediated 6β-hydroxylation of testosterone was, therefore, masked by UGT and 5α and 5β-reductase activities otherwise the difference between HH and HLM metabolism-rates would be much higher. Therefore, varying extents of contribution from non-CYP metabolism produce a range of masking-effects reflected in the HLM/HH CL_int,u_ ratios with the highest disconnect observed in compounds such as vinorelbine with no evidence of non-CYP metabolism^43,45^. Thus, consistent with our previous conclusion^21^, the intrinsic HH CYP3A4 activity appears lower relative to NADPH-HLM. In addition, the HLM to HH disconnect may be more prevalent than was at first thought.

### Lower HH CYP3A4 activities relative to NADPH-HLM can manifest as disparate CYP phenotyping

**Fig. 2:**
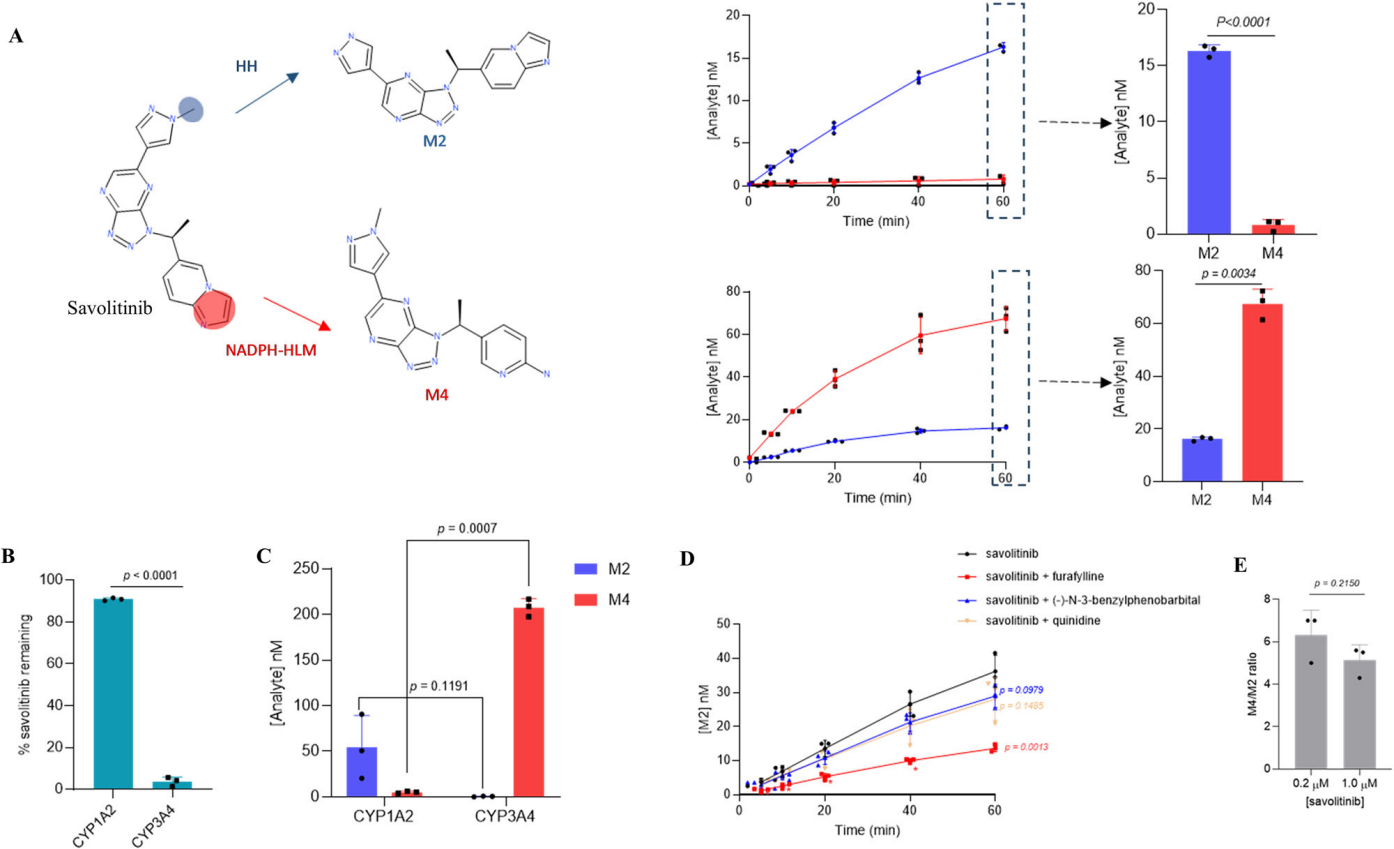
Lower HH CYP3A4 activities relative to NADPH-HLM can manifest as disparate CYP phenotyping. **A** Relative levels of savolitinib metabolites M2 and M4 formed over 1 h by HH and NADPH-HLM after incubation of savolitinib (1 µM) including a comparison of M2 and M4 levels at the 60 min time point. The data represents the mean +SD of three independent determinations and statistical significance was assessed by the unpaired two-tailed *t*-test with Welch’s correction. **B** Comparison of the percentage savolitinib remaining after incubating with recombinant CYP1A2-POR (CYP:POR ratio of 1:4.4) and CYP3A4-POR (CYP:POR ratio of 1:4), final CYP concentration in incubation of 100 pmol/mL for 1 h. The data represents the mean +SD of three independent determinations and statistical significance was assessed by the unpaired two-tailed *t*-test with Welch’s correction. **C** Comparison of M4 and M2 concentrations after incubating with recombinant CYP1A2-POR and CYP3A4-POR enzymes for 1 h. The data represents the mean +SD of three independent determinations and statistical significance was assessed by the unpaired two-tailed *t*-test with Welch’s correction. **D** Effect of specific inhibitors (furafylline, 20 µM, (-)-N-3-benzylphenobarbital, 1 µM) and quinidine, 10 µM) on formation of M2 after incubating savolitinib (1 µM) over 1 h in HH. The data are plotted as the average +SD of three independent determinations. Statistical significance was determined using multiple unpaired t-tests corrected for multiple comparisons using the Holm-Sidak method. Asterisks show statistical significance between savolitinib and savolitinib + furafylline at each time point with the *p* value for the 60 min time point stated for all inhibitors versus savolitinib alone. **E** Effect of savolitinib concentration (0.2 µM and 1.0 µM) on the savolitinib metabolite ratios (M4:M2) determined in NADPH-HLM at 37 °C for 1 h. The data represents the mean +SD of three independent determinations and statistical significance was assessed by the unpaired two-tailed *t*-test with Welch’s correction.

Human [^14^C] excretion and metabolism studies have shown that savolitinib is metabolised via *N*-demethylation to the major metabolite M2, accounting for 60% of the dose in healthy volunteers^52^. Oxidation by aldehyde oxidase accounts for 19% of the dose, while oxidation/hydrolysis on the imidazopyridine moiety makes up 16% of the dose. In this study and consistent with in vivo data, M2 was the only peak, by UV detection, present at greater than 1% after savolitinib (5 µM) was incubated with HH for 1 h. This was confirmed using authentic standards of M2 and M4, one of the products of metabolism on the imidazole ring (Fig. 2A). In contrast to *N*-demethylation in HH, opening of the imidazole ring was the major route of metabolism in HLM (Fig. 2A). Consequently, unlike HH, the HLM metabolite profile for savolitinib was not consistent with the observed in vivo human metabolite profile. In addition, incubations of savolitinib in a panel of drug metabolising recombinant CYP-POR systems identified CYP3A4 as the major metabolising CYP (Supplementary Table 1) with almost complete disappearance of savolitinib after a 60 min incubation (Fig. 2B). Surprisingly, CYP3A4 did not form M2 (Fig. 2C), the major metabolite in HH and in vivo. CYP1A2 contributed very little towards savolitinib metabolism relative to CYP3A4 in recombinant CYP-POR systems, with over 90% of savolitinib remaining after a 60 min incubation (Fig. 2B). It was, therefore, puzzling that CYP1A2 was found to be the major contributor to formation of M2 (Fig. 2C). Furthermore, specific inhibitors (Fig. 2D) confirmed CYP1A2 (inhibited by furafylline) as the major savolitinib-metabolising CYP in HH with minor contributions by CYP2D6 (inhibited by quinidine) and CYP2C19 (inhibited by (-)-N-3-benzylphenobarbital). NADPH-HLM and recombinant CYP-POR systems, both incorrectly identified CYP3A4 as the major savolitinib-metabolising CYP. In contrast, HH identified CYP1A2 as the major metabolising CYP.

It is well-known that poor passive permeability can limit the rate of metabolism in HH^12^. However, savolitinib showed high passive permeability in Caco-2 cells (*P*_app_ = 53.7 × 10^−6 ^cm/s) and high free fraction in both HLM and HH (93.5 and 84.7%, respectively), thus, the lower HH metabolism-rate was unlikely to be permeability or binding-limited. To explore whether possible enzyme kinetics favouring M2 over M4 formation at lower savolitinib concentrations could explain the observed discrepancy in M4:M2 metabolite ratios between HH and HLM, the ratio of M4:M2 was assessed in NADPH-HLM at 1 µM and 5-fold lower concentration (0.2 µM) and showed no change in the ratio (Fig. 2E) thus supporting the notion that the intrinsic CYP3A4 activity in HH is fundamentally lower relative to NADPH-HLM.

### HH CYP3A4-mediated midazolam intrinsic clearance is increased by gefitinib and recapitulated in NADH-HLM but not canonical NADPH-HLM

**Fig. 3:**
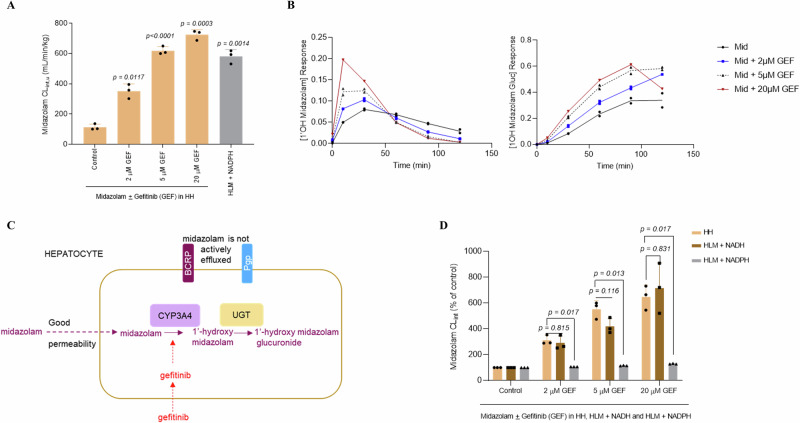
HH CYP3A4-mediated midazolam intrinsic clearance is increased by gefitinib and recapitulated in NADH-HLM but not canonical NADPH-HLM. **A** The effect of increasing concentrations of gefitinib on the scaled CL_int,u_ of midazolam, the prototypic substrate of cytochrome P450 3A4 in HH and how this compares with the scaled CL_int,u_ in NADPH-HLM. The data represents the mean + SD of three independent replicates. Statistical significance was assessed by the Brown-Forsythe and Welch ANOVA followed by Dunnett’s T3 multiple comparison of the control to the other groups. **B** Effect of increasing concentrations of gefitinib on formation of the primary metabolite (1´-hydroxy midazolam) and its secondary metabolite (1´-hydroxymidazolam glucuronide) expressed as response ratio (peak area/internal standard). The data represents a single determination. **C** Schematic view of the entry of midazolam and gefitinib into an intact hepatocyte and effect of gefitinib on the subsequent well-established midazolam sequential metabolism^46,75^ – effect of gefitinib occurs at the level of formation of 1´-hydroxymidazolam. **D** The ability of gefitinib to elicit the same effect on midazolam scaled CL_int,u_ in HH relative to NADH-HLM or NADPH-HLM. The data are from three independent determinations expressed as a percentage of the control (without gefitinib, set to 100%). At each concentration of gefitinib, statistical significance was assessed by the Brown-Forsythe and Welch ANOVA followed by Dunnett’s T3 multiple comparison of the gefitinib effect on HH midazolam scaled CL_int,u_ to the NADH-HLM and NADPH-HLM groups.

Metabolism of the prototypical CYP3A4-substrate midazolam is well-characterised and shows CYP3A4 as the major metabolising enzyme in both HH and HLM^46^. Here, midazolam exhibited over 5-fold higher HLM scaled CL_int,u_ relative to HH when NADPH was used as cofactor (i.e. control vs HLM-NADPH, Fig. 3A) which was consistent with previous studies that have demonstrated differences in rates of midazolam metabolism between HH and HLM^16,53,54^. Although midazolam is not a substrate of active efflux as shown by our previous study in the typical assay system utilising Caco-2 cells^21^, we decided to confirm this in HH themselves and, therefore, co-incubated midazolam with increasing concentrations of gefitinib, an inhibitor of the efflux transporter proteins BCRP and Pgp^55,56^. Surprisingly, gefitinib increased the HH midazolam scaled CL_int,u_ in a concentration-dependent manner with over 6-fold increase at the highest concentration tested of 20 µM (Fig. 3A) which was accompanied by increases in levels of 1´-hydroxymidazolam and the secondary 1´-hydroxymidazolam glucuronide (Fig. 3B). Understanding possible mechanisms underlying the remarkable increase in midazolam scaled CL_int,u_ caused by gefitinib was critical as this would likely provide clues on the intracellular dynamics underpinning the lower intrinsic CYP3A4 activities in HH relative to NADPH-HLM. To further rule out the possible involvement of active efflux, we measured the efflux ratio in the MDCKII-MDR1-BCRP cell line over-expressing both Pgp and BCRP, with the latter expressed at higher level relative to Pgp^57^. In addition, the high-Pgp expressing human National Institutes of Health (NIH) MDCKI-MDR1 was also utilised^58^. The results showed that midazolam at the 0.1 µM used to screen compounds for possible active efflux at AstraZeneca, is unlikely to be a substrate of BCRP or Pgp (Supplementary Table 2). We reasoned that the midazolam-gefitinib interaction likely occurred at the level of the enzyme (Fig. 3C) and if that was the case, would be reproducible in HLM. The gefitinib-effect could not, however, be reproduced in NADPH-HLM (Fig. 3D). We hypothesised that Cyt*b*_*5*_R, not POR, could be important in HH and to explore this, replaced NADPH with Cyt*b*_*5*_R-dependent NADH in HLM. Strikingly, NADH-HLM recapitulated the gefitinib-mediated increase in midazolam metabolism observed in HH (Fig. 3D).

### Replacing NADPH with NADH as cofactor in HLM produces rates of metabolism that are more representative of HH

Given the recapitulation of the HH midazolam-gefitinib interaction in NADH-HLM (Fig. 3D), we next compared the scaled CL_int_,_u_ values in HH for 64 compounds from the literature and across AstraZeneca projects, with those determined in HLM supplemented with either NADH or NADPH. The compounds represented the three general classes: acids, bases and neutrals (Supplementary Table 3) with molecular weights ranging from 179 to 721 and logD pH 7.4 values (between <1 and 4.25). The scaled CL_int,u_ values covered a wide range from very low (<10 mL/min/kg) to high (just over 1000 mL/min/kg) in both models. The intrinsic permeabilities as assessed in the Caco-2 cell line also covered a wide range (*P*_app_ values < 1–82 × 10^−6 ^cm/s). It is important to point out that the pooled HH and HLM used in this study undergo stringent in-house validation with respect to enzyme activities and these fall within literature values^9,12,16,53,54,59^.

**Fig. 4:**
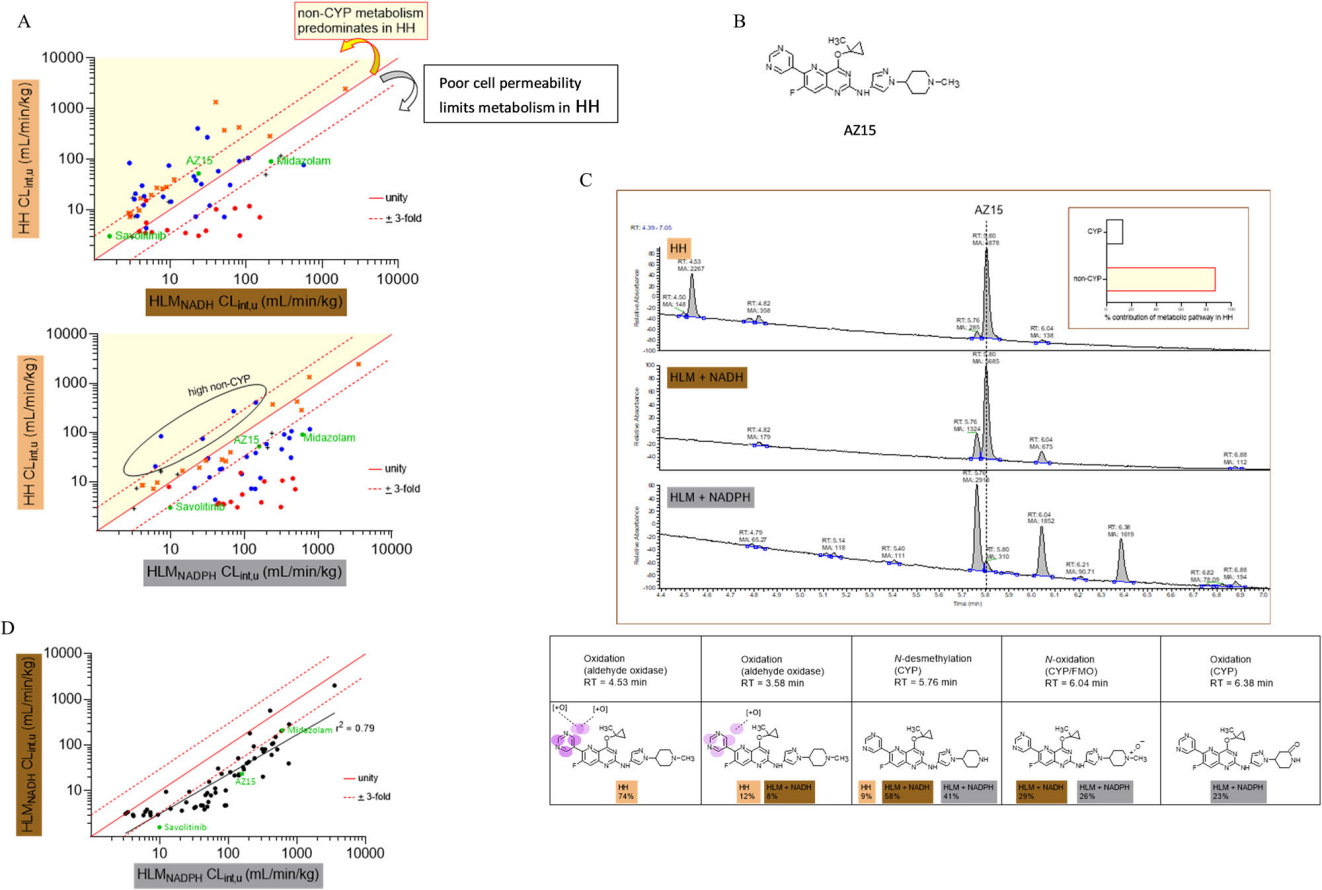
Replacing NADPH with NADH as cofactor in HLM produces rates of metabolism that are more representative of HH. **A** Comparison of scaled CL_int,u_ values for 64 literature and AstraZeneca compounds determined in HH and in HLM using NADH or NADPH as cofactor. The fraction unbound used to calculate the scaled CL_int,u_ was measured or estimated by in silico models of Fu_inc_^76^. Each point represents the scaled CL_int,u_ for an individual compound from a single determination highlighting compounds with good permeability in Caco-2 cells *P*_app_ > 7 × 10^−6 ^cm/s (blue dots), compounds with poor permeability in Caco-2 cells, *P*_app_ < 0.5 × 10^−6 ^cm/s (red dots), permeability could not be determined for 11 compounds (black cross), the scaled CL_int,u_ values for AZ compound (AZ15), savolitinib and the prototypic cytochrome P450 3A4 substrate midazolam all with good cell permeability (green dots), the major nonCYP3A4 substrates i.e. CYPs 1A2, 2C8, 2C9, 2C19 and 3A5 (orange crosses). The solid red line represents the line of unity and the dotted lines, the three-fold difference. Compounds with non-CYP metabolic pathways predominating in HH would be expected to lie in the region highlighted in yellow and those with metabolism-limiting permeability in white. **B** Structure of AstraZeneca compound AZ15. **C** A comparison of metabolite profiles (UV chromatograms and insert showing relative contributions of CYP versus non-CYP metabolism) after incubating 5 µM of AZ15 in HH and HLM with NADH or NADPH as cofactor for 1 h at 37 °C. The metabolites at retention times (4.53 and 3.58) are products of the cytosolic aldehyde oxidase (some cytosol ‘contaminating’ or carried over into microsomes during preparation by centrifugation yields the aldehyde oxidase metabolite in the NADH and NADPH profiles). Also shown as part of **C** are the structures of metabolites (products of oxidation by aldehyde oxidase and CYP, N-demethylation by CYP) that together, account for over 90% of metabolism in the respective matrices. **D** A comparison of the scaled CL_int,u_ values between HLM with NADH and NADPH as cofactors. The solid black line represents the line of regression ($y=0.86x-0.34$, r^2^ = 0.79, *P* < 0.0001).

For HH and HLM possessing consistent CYP activities, higher rates of metabolism in HLM would be anticipated for compounds with poor membrane permeability. While active efflux would be an additional important contributor to lower than expected intracellular concentrations, it is not clear whether active efflux occurs at all in suspended HH^7^. On the other hand, active uptake and/or significant non-CYP metabolism would be expected to drive higher rates of metabolism in HH relative to HLM. Both models would otherwise be expected to give similar scaled CL_int,u_ values within experimental error. Consistent with previous data from our group^41^, HLM supplemented with NADPH exhibited good concordance with HH for substrates of CYPs (Fig. 4A excluding CYP3A4, orange crosses). The rest of the compounds with significant CYP3A4 metabolism and varying extents of nonCYP metabolism showed more HH-consistent metabolism with NADH relative to NADPH under the experimental conditions in this study (Fig. 4A). A detailed examination of the metabolites produced for one of the compounds showed NADH as more representative of HH versus NADPH (Fig. 4C).

We recently demonstrated cytosolic aldehyde oxidase (AO) as an important contributor to metabolism of the 5-azaquinazoline series of IRAK-4 inhibitors^21^. Detailed metabolite identification of a compound from this series (AZ15, Fig. 4B) showed oxidation by AO as the main route of metabolism in HH with very little contribution from CYPs (Fig. 4C). A higher scaled CL_int,u_ would, therefore, be expected in HH relative to HLM and a comparison of the AZ15 HH scaled CL_int,u_ and metabolite profile showed concordance with that of NADH-HLM. In contrast, NADPH-HLM exhibited a much higher rate of metabolism characterised by an almost complete disappearance of the parent compound (AZ15) accompanied by high levels of primary metabolites and some secondary metabolites not observed in HH.

A comparison of the two cofactors in HLM showed a systematic difference of around 3-fold higher scaled CL_int,u_ values in NADPH relative to NADH (r^2^ = 0.79, Fig. 4D). Interestingly, for 10% of the compounds, both cofactors produced similar scaled CL_int,u_ values.

### NADPH and NADH are present at equivalent concentrations in hepatocyte cytosol suggesting cellular conditions render CYP redox-partner selection cofactor-independent

**Fig. 5:**
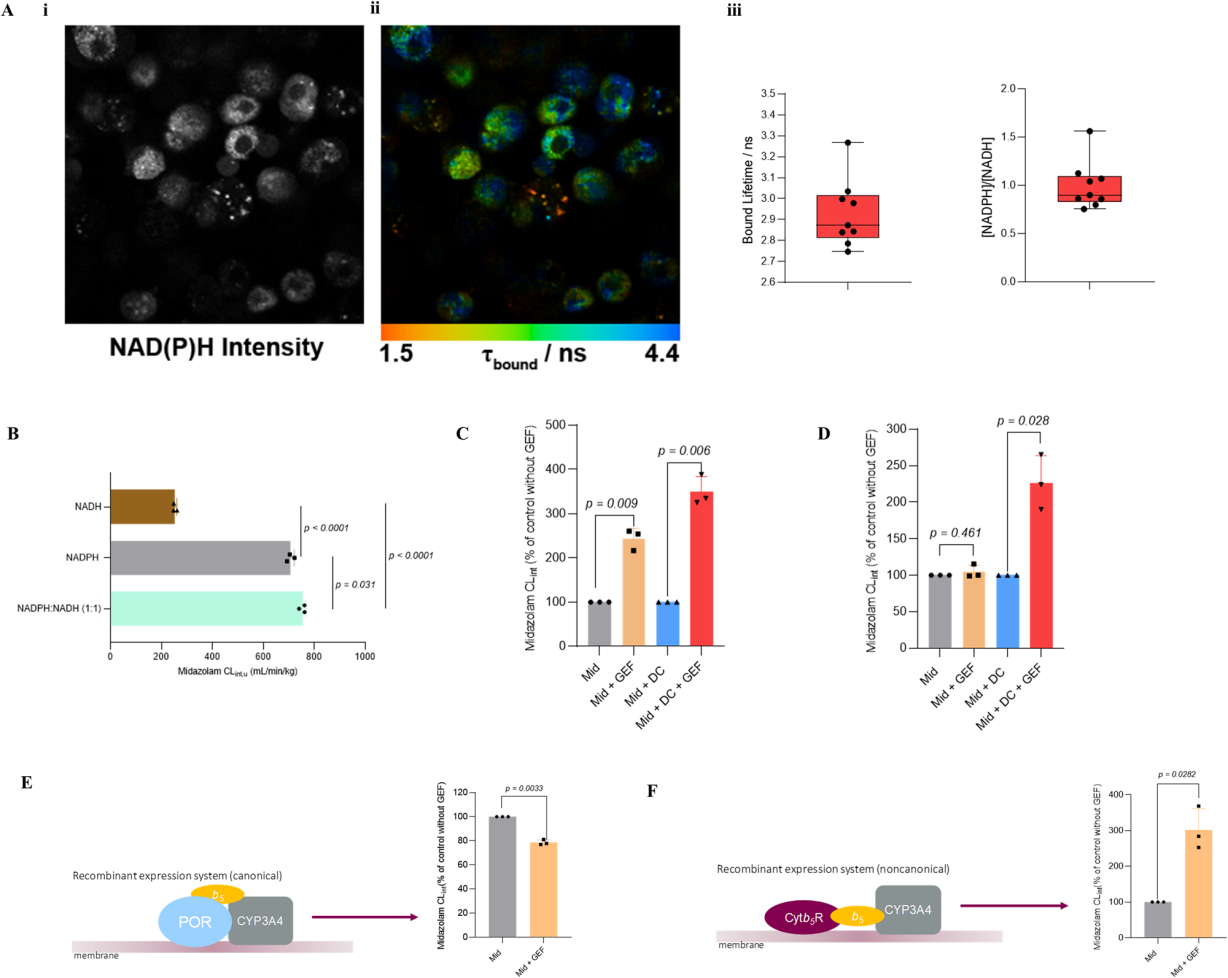
NADPH and NADH are present at equivalent concentrations in hepatocyte cytosol suggesting cellular conditions render CYP redox-partner selection cofactor-independent. **A i** Live cell fluorescence lifetime imaging (FLIM) showing images of lifetime distribution in single hepatocytes. Mean ԏ_bound_ lifetimes have been colour coded as indicated and are plotted in **ii,** the ratio of NADPH/NADH calculated from the FLIM data in HH cytosol are plotted in **iii**. The data represents the mean of three readings each from three separate vials of HH. **B** comparison of midazolam scaled CL_int,u_ in HLM with NADH, NADPH or mixture of NADPH:NADH (1:1). The data is presented as the mean +SD of three independent replicates. Statistical significance was assessed by the Brown-Forsythe and Welch ANOVA followed by Dunnett’s T3 multiple comparison of all groups. Effect of gefitinib (GEF, 5 µM) on the scaled CL_int,u_ of midazolam (Mid, 1 µM) alone or in the presence of the NADPH P450 oxidoreductase inhibitor diphenyleneiodonium chloride (DC, 5 µM) determined in HLM (1 mg/mL) fortified with **C** NADH or **D** NADPH as cofactor. The data are expressed as a percentage of the control (Mid or Mid + DC, set to 100%) represents the mean + SD of three independent determinations and statistical significance was assessed by the unpaired two-tailed *t*-test with Welch’s correction. **E** Effect of gefitinib (5 µM) on the midazolam CL_int.u_ by recombinant human CYP3A4-POR containing cytochrome *b*_*5*_. The data are expressed as a percentage of the control (Mid set to 100%), represents the mean + SD of three independent determinations and statistical significance was assessed by the unpaired two-tailed *t*-test with Welch’s correction. **F** Effect of gefitinib (5 µM) on the midazolam CL_int.u_ by recombinant human CYP3A4-Cyt*b*_*5*_R containing cytochrome *b*_*5*_. The data are expressed as a percentage of the control (Mid set to 100%), represents the mean + SD of three independent determinations and statistical significance was assessed by the unpaired two-tailed *t*-test with Welch’s correction.

While exogenous cofactors are required by cell-free CYP models including HLM and recombinant systems, the cofactors cannot permeate the plasma membrane of intact cells. Therefore, intact cells rely on cofactor pools generated intracellularly. Based on HLM data, for Cyt*b*_*5*_R to be the main CYP redox partner in HH, the intracellular concentrations of NADH would not only have to be much higher than NADPH, but the NADPH concentration must be much lower than the POR K_m_ value. Both NADH and NADPH are fluorescent, allowing measurements of the reduced state of the cofactors in intact cells^60^. However, distinguishing fluorescence between the two cofactors in the same sample is a key challenge as their fluorescence spectra are identical. This was resolved using fluorescence lifetime imaging, FLIM, as previously described^61^. The fluorescence lifetimes of NADH and NADPH when enzyme-bound are distinct, allowing calculation of the relative levels of the two cofactors based on the mean bound lifetimes measured^61^. These data revealed that the two cofactors are present at equivalent concentrations in HH cytosol (Fig. 5A) and this is consistent with literature reports reviewed by Li et al.^62^ showing free cytosolic NADPH concentrations of 3 µM with similar NADH levels expected given NAD^+^ concentrations of up to 110 µM (NAD^+^ concentrations can be 60-fold higher than NADH).

Using midazolam as substrate, an equimolar combination of the cofactors in HLM gave similar reaction rates to NADPH alone (Fig. 5B) indicating that in the presence of both cofactors, POR is the dominant electron donor and NADH does not interfere or inhibit POR under the HLM experimental conditions. For Cyt*b*_*5*_R-dependent CYP3A4 catalysis to occur, we reasoned that cellular conditions in which the cofactor levels are similar, would have to render CYP redox-partner selection cofactor-independent, in a way that is analogous to inhibiting CYP interactions with POR. To explore this, we inhibited POR using diphenyleneiodonium chloride (DC) which acts by irreversibly binding, in a time-dependent manner, to the FMN moiety with a *K*_*i*_ of 2.8 mM^63^. Although DC is an inhibitor of other flavin-containing enzymes and, therefore, likely to inhibit Cyt*b*_*5*_R, its mechanism of inhibition requires reduction of the flavin moiety before it can bind irreversibly. We reasoned that POR, with its higher affinity for NADPH, would more readily reduce its flavin moiety and be susceptible to greater inhibition relative to Cyt*b*_*5*_R especially with the much lower concentration of diphenyleneiodonium chloride (5 µM) relative to the reported *K*_*i*_ and short pre-incubation time (5 min). Intriguingly and in line with its effect in the non-canonical NADH-HLM (Figs. 3A and 5C), gefitinib increased the midazolam intrinsic clearance in canonical NADPH-HLM in the presence of DC (Fig. 5D), suggesting that under these experimental conditions, Cyt*b*_*5*_R becomes the main electron donor to CYP3A4 and can utilise NADPH in place of NADH.

To validate the key finding that Cyt*b*_*5*_R is important for HH CYP3A4-catalysed reactions as indicated by the gefitinib-mediated increase in CYP3A4-mediated midazolam intrinsic clearance in Cyt*b*_*5*_R- and not POR-dependent HLM (Figs. 3A, D and 5C, D), we ordered a custom synthesis of non-canonical recombinant CYP3A4-Cyt*b*_*5*_R with cytochrome *b*_*5*_. We then assessed the impact of gefitinib on the CYP3A4-Cyt*b*_*5*_R or CYP3A4-POR midazolam CL_int_. Intriguingly, gefitinib inhibited the metabolism of midazolam in CYP3A4-POR (Fig. 5E). In contrast and in line with HH, NADH-HLM and POR-inhibited (by DC) NADPH-HLM, gefitinib increased the midazolam intrinsic clearance in incubations with NADPH-supplemented CYP3A4-Cyt*b*_*5*_R (Fig. 5F).

### Mimicking the dense intracellular protein renders CYP redox-partner selection cofactor independent and recapitulates HH savolitinib-metabolism

**Fig. 6:**
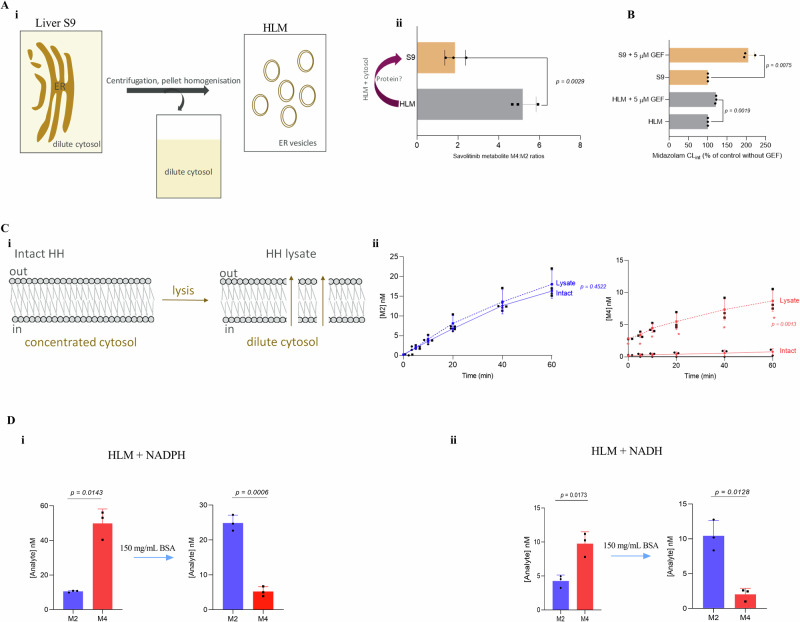
Mimicking the dense intracellular protein renders CYP redox-partner selection cofactor independent and recapitulates HH savolitinib metabolism in HLM. **A i** HLM are prepared by re-suspending the pellet obtained from liver S9 fraction by centrifugation at 100,000 × *g*. The absence of cytosol, therefore, distinguishes HLM from liver S9. **ii** Comparison of savolitinib metabolite ratios (M4:M2) after incubating savolitinib (1 µM) at 37 °C for 1 h in human liver S9 and microsomes from the same pool of donors supplemented with NADPH. The data represent an average +SD of three independent duplicate measurements and statistical significance was assessed by the unpaired two-tailed *t*-test with Welch’s correction. **B** comparison of the effect of gefitinib (5 µM) on midazolam (1 µM) CL_int,u_ in liver microsomes and liver S9 from the same pool of donors fortified with NADPH incubated at 37 °C for 1 h, in each case, the control was set to 100%. The data represent an average +SD of three independent duplicate measurements and statistical significance was assessed by the unpaired two-tailed *t*-test with Welch’s correction. **C i** Lysis of HH (preparation described in materials and methods) results in dilution of the cell’s contents including cytosol and cofactors. **ii** Effect of HH lysis on the formation of savolitinib metabolites M4 and M2 after incubating savolitinib (1 µM) in intact HH and HH lysate (supplemented with NADPH, details in materials and methods). The data are expressed as the mean of three determinations from three different vials of HH. Statistical significance was determined using multiple unpaired *t*-tests corrected for multiple comparisons using the Holm-Sidak method. Asterisks show statistical significance between intact and lysed HH at each time point with the *p* value for the 60 min time point stated for both metabolites. **D** Effect of bovine serum albumin (final 150 mg/mL) on the relative levels of savolitinib metabolites M2 and M4 formed in human liver microsomes fortified with **i** NADPH or **ii** NADH at 37 °C for 1 h. The data represent an average +SD of three independent duplicate measurements and statistical significance was assessed by the unpaired two-tailed *t*-test with Welch’s correction.

As HLM are vesicles of the endoplasmic reticulum, a reasonable first step of exploring for possible intracellular factors that could drive the cofactor-independent selection of CYP redox partnerships would be the addition of cytosol to HLM i.e. the liver S9 fraction. The absence of cytosol distinguishes HLM from liver S9 (Fig. 6A, i). For this to be viable, the S9 and HLM would have to be from the same donor to ensure the same enzyme expression levels. The ratio of savolitinib metabolites M4:M2 (Fig. 2A) provided an excellent probe in the search for possible intracellular factors. The M4/M2 ratio was, therefore, measured in HLM and liver S9 supplemented with NADPH. Intriguingly, the ratio of M4:M2 was significantly different with a shift towards HH ratios in liver S9 (Fig. 6A, ii). A component of cytosol, therefore, caused the metabolite ratio shift and, if in line with the notion that Cyt*b*_*5*_R is important for HH CYP3A4 activity, then more Cyt*b*_*5*_R-CYP3A4 interactions would be expected in S9 relative to HLM. By extension, gefitinib would be expected to produce a higher increase in midazolam CL_int,u_ in liver S9 relative to HLM and would be in line with observations in Fig. 5 supporting the notion that the increase in midazolam caused by gefitinib only occurs when Cyt*b*_*5*_R is the CYP3A4 redox partner. Indeed, gefitinib caused a much higher (two-fold) increase in midazolam CL_int,u_ in S9 relative to HLM from the same donor pool (Fig. 6B).

Given that the cytosol in liver S9 is significantly more dilute relative to intact cells (the preparation of liver S9 involves homogenisation steps in buffers that result in dilution of the cytosol), we reasoned that a component had to be present at high concentrations in intact cells to cause the notable decrease in M4:M2 ratios in the dilute cytosol in liver S9 relative to HLM (Fig. 6A, ii). A further test of this notion could be achieved via lysis of HH – the resultant dilution of the cytosol (Fig. 6C, i) would be expected to shift the M4:M2 ratio towards that observed in HLM i.e. formation of more M4. Indeed, M4 which was barely formed in intact HH, was significantly increased but not M2 (Fig. 6C, ii). As protein-protein interactions between reductases and CYPs are necessary for electron transfer to occur, the presence of other proteins is more likely to modify these interactions relative to small molecules. We, therefore, decided to explore the possible impact of intracellular protein concentrations which can be as high as 300 mg/mL in an intact cell^64^. In comparison, typical in vitro HLM incubation conditions utilise protein concentrations around 1 mg/mL representing ~300-fold dilution over HH. Interestingly, a shift towards HH savolitinib M4:M2 ratio was observed with increasing concentrations of cytosol added to NADPH-HLM (Supplementary Fig. 1). Achieving cytosolic protein concentrations in the range of those in intact cells, in typical incubations in vitro would be virtually impossible. Previous reports show that proteins such as bovine serum albumin can be used to mimic the high cytosol protein concentrations^64^. Such an experiment would likely be viable for a compound that is not highly protein bound. A final concentration of 150 mg/mL would be in the range of intracellular cytosolic protein concentrations and allow for sufficiently free (17%) savolitinib to monitor its metabolism with the extent of binding consistent with the 29% free^65^ in normal plasma (which has ~3-fold lower albumin). Critically, at this concentration, we established that the kinetics of M4 and M2 formation would not be impacted by the lower concentration (Fig. 2E). Intriguingly, when the NADPH-HLM incubation conditions were modified to mimic the intracellular protein by adding BSA (final concentration of 150 mg/mL), the metabolism switched from the CYP3A4 dominated M4 formation to phenocopy the HH M2 predominantly mediated by CYP1A2 as the major route of metabolism (Fig. 6D i). A similar savolitinib profile was obtained in NADH-HLM (Fig. 6D ii) confirming that in the presence of both NADPH and NADH (as expected from measurements in intact HH, Fig. 5A), M2 is the major route of savolitinib metabolism. Interestingly, using savolitinib or the prototypical CYP1A2 substrate phenacetin, there was no detectable turnover with noncanonical CYP1A2- Cyt*b*_*5*_R i.e. no metabolite formation or disappearance of parent compound.

## Discussion

A sound understanding of in vitro assays that feed into in vivo candidate drug-metabolism projections builds confidence in human pharmacokinetics predictions^66^. As a result, efforts to understand the disproportionately lower scaled CL_int,u_ values in HH relative to NADPH-HLM exhibited by, mainly CYP3A4 substrates, have been a priority for researchers. Here, we show that the disconnect in CL_int,u_ values between the two models can manifest as disparate CYP phenotyping in which NADPH-HLM and recombinant CYP-POR systems incorrectly identify CYP3A4 while HH accurately assign the main-metabolising CYP. For savolitinib, we unequivocally demonstrate that, in agreement with in vivo data^52,67^, CYP1A2 is the main metabolising enzyme in HH. Critically, we show that the savolitinib CYP phenotyping discrepancy cannot be explained by differences in enzyme expression levels between the models or poor passive permeability as suggested by others^12^.

While poor passive permeability can indeed limit the rate of metabolism in HH, the high passive permeability of savolitinib, as assessed in Caco-2 cells, makes this highly unlikely. Even if another unknown mechanism produced lower intracellular savolitinib concentrations relative to HLM, this would still not explain the mismatch in CYP phenotyping because the CYP3A4 and CYP1A2 savolitinib-metabolism kinetics are not expected to be impacted by the differences in savolitinib concentration as demonstrated by the unchanged metabolite ratios in HLM. It is noteworthy that poor passive permeability as a general explanation for the discrepancy in metabolism-rates under investigation here, is comprehensively discounted as the observed increase in midazolam-metabolism rate in HH caused by gefitinib would not occur if intracellular midazolam concentrations were limiting.

Regarding enzyme expression levels, it is important to appreciate that experiments with recombinant CYP-POR enzymes were conducted using the same concentration of each CYP and it is under these conditions that savolitinib almost completely disappears in incubations with CYP3A4 while over 90% remains with CYP1A2. The striking drop in HH CYP3A4 activity as measured by the M4 concentration cannot, therefore, be explained by differences in enzyme expression levels as only almost-complete ablation of CYP3A4 expression would capture the HH savolitinib-metabolite profile. Such expression levels are highly unlikely as CYP3A4 is the most abundant liver CYP^68,69^, moreover, pooled batches of HH in use at AstraZeneca undergo extensive evaluation in collaboration with commercial suppliers to ensure representative enzyme activities. In addition, we show a shift towards more HH-like savolitinib metabolite ratios in liver S9 relative to HLM from the same pool of donors that further discounts differences in enzyme expression levels as an explanation thus supporting the notion that a fundamental difference in intrinsic CYP3A4 activities underpins the disconnect between the two models. The consistent savolitinib-metabolism between recombinant CYP-POR and another POR-centric system, NADPH-HLM, but not HH, spotlights CYP redox partnerships and whether the two in vitro systems capture redox interactions prevailing in HH.

The challenges associated with studying CYP-redox partner interactions are noteworthy^3^ and explain the lack of clarity regarding the precise contributions of POR and Cyt*b*_*5*_R to CYP metabolism. It is, however, clear that for electron transfer to occur, a complex between a CYP and POR is necessary^70^. Here, the serendipitous discovery that the HH CYP3A4-mediated midazolam clearance is increased by gefitinib and recapitulated in non-canonical Cyt*b*_*5*_R-dependent HLM and recombinant CYP3A4-Cyt*b*_*5*_R show that Cyt*b*_*5*_R could be important for HH-CYP3A4 activity. By extension, the CYP3A4-Cyt*b*_*5*_R/*b*_*5*_ complex, necessary for electron transfer to occur, likely presents interaction sites not available on CYP3A4-POR that gefitinib binds to, resulting in the activation of midazolam clearance and further studies are required to understand the exact mechanism. We show CYP3A4 activities that are more representative of HH in NADH-HLM for multiple compounds that support the notion that Cyt*b*_*5*_R is important for HH-CYP3A4 activity. We further confirm the importance of Cyt*b*_*5*_R via DC-mediated POR inhibition in NADPH-HLM that facilitates the isolation of Cyt*b*_*5*_R activity resulting in recapitulation of the gefitinib-mediated increase in midazolam intrinsic clearance. Therefore, in this work, three orthogonal methods demonstrate that the gefitinib-mediated increase in midazolam metabolism-rate only occurs when Cyt*b*_*5*_R is the redox partner for CYP3A4, strongly supporting the prevalence of CYP3A4-Cyt*b*_*5*_R interactions over CYP3A4-POR in HH. Critically, this observation is consistent with results from in vivo knockdown of Cyt*b*_*5*_R’s cognate redox partner cytochrome *b*_5_ showing cytochrome *b*_*5*_ to be important for some CYP substrates such as midazolam^38^.

The CYP1A2 activity in noncanonical NADH-HLM is too low to recapitulate HH-CYP1A2 savolitinib-metabolism and, is consistent with the failure by recombinant CYP1A2-Cyt*b*_*5*_R to metabolise savolitinib or the prototypical CYP1A2 substrate phenacetin. This suggests CYP3A4 and CYP1A2 likely utilise Cyt*b*_*5*_R and POR, respectively, a notion consistent with the observation that cytochrome *b*_*5*_ knockdown in vivo was important for some but not all substrates^38^. This, however, raises the question of how this could be possible as, under the experimental conditions utilised for HLM, redox partner selection is determined by the added cofactor. Imaging NADH/NADPH in hepatocytes showed equivalent concentrations of both cofactors suggesting that intracellular factors render CYP redox-partner selection cofactor-independent. Although cofactor levels, here measured, are relative and not absolute, we can conclude, based on the remarkable increase in midazolam clearance caused by gefitinib in HH, that the intracellular cofactor levels are not limiting.

Our first attempt at exploring possible intracellular factors responsible for imparting cofactor-independence showed a shift towards HH savolitinib-metabolite ratios in liver S9 (cytosol + HLM) relative to HLM, both prepared from the same pool of donors. This suggests that a component of cytosol promotes CYP redox partnerships prevailing in HH. In addition, we show a greater increase in midazolam clearance caused by gefitinib, in liver S9 relative to HLM which, as demonstrated above, only occurs when Cyt*b*_*5*_R is the CYP3A4 redox partner. Thus, a component of cytosol promotes more CYP3A4-Cyt*b*_*5*_R interactions in liver S9 relative to HLM. As a strategy for a more-focused search for the cytosolic component, we reasoned that protein-protein interactions are more likely modulated by other proteins and could, therefore, be the missing factor from HLM given that intracellular protein concentrations can be as high as 300 mg/mL compared to just 1 mg/mL used in typical HLM-incubations. To this end, we show a cytosolic-protein concentration-dependent shift towards HH savolitinib metabolite ratios. However, as it is virtually impossible to achieve the same intact-cell protein concentrations via addition of cytosol to HLM incubations, we show that the addition of bovine serum albumin, used by others as a surrogate^64^, can attain the range of cytosolic protein concentrations in intact cells and recapitulate the HH-savolitinib metabolism in NADPH-HLM.

Our findings provide mechanistic insights into the modulation of CYP-metabolism rates and routes via CYP-dependent redox partnerships underpinned by intracellular factors including the dense-cytosolic protein. While some CYPs such as CYP1A2 may utilise POR, our work suggests Cyt*b*_*5*_R together with cytochrome *b*_*5*_ to be key for the most important CYP, CYP3A4 in HH. For both POR- and Cyt*b*_*5*_R-dependent CYP-metabolism, intracellular factors drive appropriate redox kinetics and resultant in vivo-relevant reaction rates. Therefore, in the absence of the intracellular factors, classical NADPH-HLM will drive CYP reaction rates and routes that may be fundamentally different from HH thus explaining the observed divergent CYP activities between HH and HLM for some compounds. In our view, these findings should prompt researchers and regulatory authorities to reconsider the use of HLM and recombinant CYP-POR systems for drug metabolism studies and usher in an era of research to design in vivo-relevant in vitro tools that may include adaptations of current models to complement HH.

## Methods

### Determination of CL_int,u_ in NADH and NADPH supplemented HLM

The reaction mixture was made up of HLM (prepared from a pool of 150 donors, batch QQY from BioIVT, final 1 mg/mL protein; Ultrapool catalogue 452115 lot 38289, Corning), potassium phosphate buffer pH 7.4 (final concentration 0.1 M), NADPH/NADH (1 mM), test compound (1 µM). The final concentration of organic solvent in the mix was 0.4% (0.38% acetonitrile and 0.02% DMSO). For savolitinib experiments in Fig. 4, in the presence/absence of bovine serum albumin (BSA), BSA dissolved in water was added to give a final concentration of 150 mg/mL. The final concentration of buffer in the presence and absence of BSA was 0.1 M KPO4 pH 7.4. After a preincubation of 5 min at 37 °C, the reaction was started by adding the substrate and aliquots (25 µL) were taken at 0, 5, 10, 20, 40, and 60 min and added to ice-cold acetonitrile containing internal standard (100 µL). After centrifugation at 3000 × *g* for 10 min, the supernatant was transferred to a clean 96-well plate and diluted 6-fold with water. After mixing, 3 µL was injected into a mass spectrometer. Calculation and scaling of CL_int,u_ was done using the equation below and 45 mg of microsomal protein per gram of liver ^21^.

$${HLM\; CL}{{int}},u=\frac{\left[\frac{{ln}2}{\,}x\frac{1}{t1/2(min )}x\frac{{mL\; incubation}}{{mg\; microsomal\; protein}}x\frac{45{mg\; microsomal\; protein}}{g\;{liver}}x\frac{21\,g\;{liver}}{{kg}}\right]}{{Fuinc}}$$

### Incubations in liver S9 and HLM from the same pool of donors

Liver S9 (lot 1810001, pool of 50, Xenotech) and HLM (pool of 50, lot 1810003, Xenotech) from the same pool of donors were purchased from Xenotech. The reaction mixture was made up of HLM or S9 (final 1 mg/mL protein), potassium phosphate buffer pH 7.4 (final concentration 0.1 M), NADPH (1 mM), test compound (1 µM). The final concentration of organic solvent in the mix was 0.4% (0.38% acetonitrile and 0.02% DMSO). After a preincubation of 5 min at 37 °C, the reaction was started by adding the substrate and aliquots (25 µL) were taken at 0, 5, 10, 20, 40, and 60 min and added to ice-cold acetonitrile containing internal standard (100 µL). After centrifugation at 3000 × *g* for 10 min, the supernatant was transferred to a clean 96-well plate and diluted 6-fold with water. After mixing, 3 µL was injected into a mass spectrometer.

### Determination of CL_int,u_ in pool of human hepatocytes

Cryopreserved HH prepared from a mixed-gender pool of twenty donors (product X008000, lot EEO, BIOIVT), ten-donors (product S01205, lot MTJ, BIOIVT), ten donors (product S01205 lot LYB, BIOIVT) were utilised in the experiments^21^. Cryopreserved HH were rapidly thawed in a water bath set at 37 °C and added to Leibovitz media that had been pre-warmed in the water bath. The suspension of cells was mixed gently and centrifuged at 40 × *g* for 4 min at room temperature. Excess media was removed, and the cells were re-suspended in 5 mL of fresh Leibovitz media. The cells were counted using an automated cell counter (Cellometer). Cells with viability >80% were used in experiments to give 1 million cells/mL in a final volume of 250 µL (2.5 µL of compound +247.5 µL cell suspension) and 1 µM of test compound. An aliquot (25 µL) was taken at 0, 5, 10, 20, 40 and 60 min and added to ice-cold acetonitrile containing internal standard (100 µL). After centrifugation at 3000 × *g* for 10 min, the supernatant was transferred to a clean 96-well plate and diluted 6-fold with water. After mixing, 3 µL was injected into a mass spectrometer. Calculation of the scaled CL_int,u_ used 120 million cells per gram of liver and the equation below ^21^.

$${CL}{int},u=\frac{\left[\frac{{ln}2}{\,}x\frac{1}{t1/2(min )}x\frac{{mL\; incubation}}{1{million\; cells}}x\frac{120{million\; cells}}{g\;{liver}}x\frac{21\,g\;{liver}}{{kg}}\right]}{{Fuinc}}$$

### Effect of gefitinib on midazolam CL_int,u_

Gefitinib (working stocks dissolved in 5% DMSO and 15% acetonitrile) was added to cryopreserved HH (final concentration 1 million cells/mL) to give final concentrations of 2, 5 and 20 µM (final organic solvent concentrations of 0.16%). The control HH used for comparisons had the same final organic solvent of 0.16%. The same solutions were utilised in experiments on the effect of gefitinib on midazolam CL_int,u_ in HLM supplemented with NADH or NADPH.

### Identification of CYPs responsible for formation of M2 in HH

Savolitinib (final 1 µM) was incubated at 37 °C with human hepatocytes (final of 1 million cells/mL in Leibovitz buffer) alone and in the presence of specific inhibitors: furafylline (CYP1A2, final 20 µM), (-)-N-3-benzylphenobarbital (CYP2C19, final 1 µM) and quinidine (CYP2D6, final 10 µM), respectively, for 1 h. Aliquots were taken at 0, 5, 10, 20, 40 and 60 min and added to cold acetonitrile containing internal standard. Authentic standards (M2 and M4) were spiked into hepatocytes (at 1 million cells/mL) and used to construct standard curves. Control marker reactions (final 1 µM for each compound) specific for CYP1A2 (phenacetin to form paracetamol), CYP2C19 (S-mephenytoin to form 4-OH mephenytoin) and CYP2D6 (dextromethorphan to form dextrorphan) were incubated in the presence and absence of the specific inhibitors to confirm the expected enzyme inhibition. Aliquots were taken at 0, 5, 10, 20, 40 and 60 min and added to cold acetonitrile containing internal standard. The acetonitrile/hepatocyte mixtures were centrifuged at 3000 × *g* for 10 min and 50 µl of the supernatant was mixed with 300 µl of water and 3 µL was injected into the mass spectrometer for analysis by multiple reaction monitoring.

### Metabolism of savolitinib in lysed hepatocytes

HH in Leibovitz buffer were centrifuged at 11,250 × *g* for 5 min to pellet the cells. Taking care not to disturb the pellet, all the media was removed using a pipette. Ice-cold water was added and the mixture vortex-mixed to resuspend the cells and stored at −80 °C for 5 min followed by sonication for 10 min to lyse the cells. The resuspended lysate final volume was equivalent to a final cell concentration of 1 million cells/mL (the same concentration used for intact cells experiments) in 0.1 mM phosphate buffer pH 7.4 and NADPH (final 1 mM). After pre-incubating for 5 min at 37 °C, the reaction (metabolism of savolitinib) in the lysate was initiated by adding savolitinib (final 1 µM). Aliquots were taken at 0, 5, 10, 20, 40 and 60 min and added to cold acetonitrile containing internal standard. Authentic standards (M2 and M4) were spiked into hepatocyte lysate and used to construct standard curves. The acetonitrile/hepatocyte lysate mixtures were centrifuged at 3000 × *g* for 10 min and 50 µl of the supernatant was mixed with 300 µl of water and 3 µL was injected into the mass spectrometer for analysis by multiple reaction monitoring.

### Determination of compound free fraction in incubations

Equilibrium dialysis in HLM (1 mg/mL) and rat hepatocytes (1 million cells/mL in Leibovitz buffer as a surrogate for HH) was utilised. Rat hepatocytes were pre-treated with 1-aminobenzotriazole (final 1 mM) and incubated in a water bath for 1 h followed by addition of salicylamide (final 1.5 mM) and incubated for a further 5 min. HLM did not require any pre-treatment. Compounds (final 1 µM) were added to the hepatocyte mixture or HLM and an aliquot was taken at time (T = 0). The mixtures were dialysed against Leibovitz buffer (for hepatocytes) or 0.1 M phosphate buffer pH 7.4 (for HLM) at 37 °C for 4 h. An aliquot was taken and, after matrix-matching added to acetonitrile containing internal standard. After centrifugation (3000 × *g* for 10 min), the supernatant was diluted 6-fold with water and 3 µL was injected into the mass spectrometer for analysis^21^.

### Reporting summary

Further information on research design is available in the Nature Portfolio Reporting Summary linked to this article.

## Incubations with recombinant human CYPs

### Screen to identify CYPs metabolising savolitinib (Supplementary Table 1)

The reaction mixture consisted of recombinant enzyme from BioIVT, originally Cypex (final 100 pmol/mL) from bactosomes prepared from *Escherichia coli*, potassium phosphate buffer pH 7.4 (final concentration 0.1 M), NADPH (1 mM) and test compound (1 µM). The reaction was started by addition of test compound after a pre-incubation of 5 min at 37 °C. Aliquots (25 µL) were taken at 0, 5, 10, 15 and 25 min and and added to ice-cold acetonitrile containing internal standard (100 µL). After centrifugation at 3000 × *g* for 10 min, the supernatant was transferred to a clean 96-well plate and diluted 6-fold with water. After mixing, 3 µL was injected into a mass spectrometer. The panel of enzymes included CYPs 1A2 (CYP001, Lot C1A2R010C), 2A6 (CYP011, Lot C2A6R008D), 2B6 (CYP020, Lot C2B6R046), 2C8 (CYP017, Lot C2C8R005), 2C9 (CYP019, Lot C2C9H028/A), 2C19 (CYP008, Lot C2C19R016D), 2D6 (CYP007, Lot C2D6R030), 2E1 (CYP009, Lot C2E1R017), 3A4 (CYP002, Lot C3A4R046B) and 3A5 (CYP046, Lot C3A5R004). The intrinsic clearance of savolitinib was calculated from the peak area determined by LC-MS/MS. The average intrinsic clearance was used from three independent determinations. For each CYP, the percentage contribution to the total metabolism of savolitinib was calculated by using the intersystem extrapolation factors and abundance of the CYP^71,72^.

For detailed comparisons between recombinant CYP1A2 and CYP3A4 (Fig. 2B, C), Supersomes were used (CYP1A2, catalogue number 456203, CYP3A4, catalogue number 456202). The reaction mixture was made up of the CYP (final 100 pmol/mL), savolitinib (1 µM), potassium phosphate buffer pH 7.4 (final concentration 0.1 M) and NADPH (1 mM). The reaction was started by addition of savolitinib after a pre-incubation of 5 min at 37 °C. An aliquot (25 µL) was added to ice-cold acetonitrile containing internal standard (100 µL). After centrifugation at 3000 × *g* for 10 min, the supernatant was transferred to a clean 96-well plate and diluted 6-fold with water. After mixing, 3 µL was injected into a mass spectrometer. The concentration of savolitinib metabolites M2 and M4 formed were determined from standard curves obtained by spiking the appropriate concentrations of M2 and M4 into recombinant enzymes (in the absence of NADPH) and processing in the same way as the samples.

### Non-canonical CYP3A4-Cyt*b*_*5*_R and CYP1A2-Cyt*b*_*5*_R

As commercial recombinant human CYPs co-express the individual CYP and reductase with an option to include cytochrome *b*_*5*_. A custom synthesis co-expressing CYP3A4 or CYP1A2 and Cyt*b*_*5*_R plus cytochrome *b*_*5*_ was, therefore, ordered from BioIVT and the bactosomes were compared with canonical CYP-POR enzymes plus cytochrome *b*_*5*_. CYP3A4-Cyt*b*_*5*_R plus cytochrome *b*_*5*_ (CYP3A4b5R bactosomes, catalogue number CE00001(CYP3A4), batch number CPD034-3A4) with a CYP concentration: 4.7 nmol/mL, cytochrome *b*_*5*_ concentration: 23.4 nmol/mL, protein concentration: 15.4 mg/mL, specific CYP content: 305 pmol/mg and NADH cytochrome c reductase activity: 208 nmol/min/mg. CYP1A2-Cyt*b*_*5*_R plus cytochrome *b*_*5*_ (CYP1A2b5R bactosomes, catalogue number CE00001 (CYP1A2), batch numberCPD034-1A2-30 with a CYP concentration: 4.7 nmol/mL, cytochrome *b*_*5*_: 23.4 nmolml, protein concentration: 16.1 mg/mL, specific CYP content: 292 pmol/mg protein, NADH cytochrome c reductase activity: 118 nmol/min/mg protein.

### Canonical CYP3A4-POR

Human CYP3A4BR EasyCYP bactosomes, catalogue number CYP/EZ005, batch number C3A4BR054, CYP concentration: 1 nmol/mL, cytochrome *b*_*5*_ concentration: 5 nmol/mL, protein: 10 mg/mL, specific CYP content: 100 pmol/mg protein, cytochrome c reductase 544 nmol/min/mg protein (1.28 nmol/mL cytochrome c reductase; CYP:POR ratio of 1:1.28).

### Comparisons of midazolam CL_int_ in the presence and absence of gefitinib between canonical and noncanonical CYP3A4

To get a measurable CL_int_ for CYP3A4-Cyt*b*_*5*_R, a final protein concentration of 1 mg/mL was incubated with potassium phosphate buffer pH 7.4 (final concentration 0.1 M), NADPH (1 mM) and midazolam (1 µM) in the presence or absence of gefitinib (5 µM). The reaction was started by addition of test compound after a pre-incubation of 5 min at 37 °C. Aliquots (25 µL) were taken at 0, 5, 10, 20, 40, and 60 min and was added to ice-cold acetonitrile containing internal standard (100 µL). After centrifugation at 3000 × *g* for 10 min, the supernatant was transferred to a clean 96-well plate and diluted 6-fold with water. After mixing, 3 µL was injected into a mass spectrometer. For CYP3A4-POR, to ensure the same free gefitinib in the incubation as for CYP3A4-Cyt*b*_*5*_R, the same final protein concentration of 1 mg/mL was used. As the reaction-rate was much higher, aliquots were taken at 0, 1, 2, 3, 4 and 5 min.

### Determination of intrinsic permeability

The intrinsic permeability of savolitinib was measured in Caco-2 cells in which efflux transporters were inhibited. Compound (10 µM) was added to the apical side in the presence of the P-glycoprotein inhibitor quinidine (50 µM), MRP2 inhibitor benzbromarone (30 µM) and breast cancer resistance protein inhibitor sulfasalazine (20 µM) and incubated at 37 °C. Aliquots were taken at 0 and 2 h and added to acetonitrile containing internal standard. After centrifugation, the supernatant was analysed by LC-MS/MS. The apparent permeability (P_app_) was calculated as follows:

$${Papp}=\frac{{VA}}{{Area\; x\; time}}x\frac{\left[{drug}\right]{acceptor}}{\left[{drug}\right]{initial},{donor}}$$

Where VA = volume (mL), Area = surface area of membrane and time (incubation time in seconds)^21^.

### Determination of LogD pH 7.4

The octanol/phosphate buffer (pH 7.4) was used to determine the LogD. Equal volumes of octanol (saturated with phosphate buffer pH 7.4) and phosphate buffer pH 7.4 (saturated with octanol) were added to 96 deep-well plates. After adding compound (dissolved in DMSO, final 1% v/v), the plates were sealed and vortex-mixed for 30 min. Equilibration for 3 h on a horizontal shaker was followed by centrifugation (3220 × *g* for 30 min). Samples from both phases were analysed by LC-MS/MS and the LogD was calculated as log of [compound] in octanol/[compound] in phosphate buffer^73^.

### Determination of efflux ratio in MDCKII-MDR1-BCRP and NIH MDCKI-MDR1 cell lines

The MDCKII-MDR1-BCRP^57^ and NIH MDCKI-MDR1^58^ cell lines were used to assess efflux. After confirmation of cell monolayer integrity, compounds were dissolved in Hank’s balanced salt solution (HBSS) pH 7.4 to achieve appropriate concentrations (final 0.1 µM). Transport assays were done by adding the working solution to the apical chamber (to assess A-B transport) and to the basolateral chamber (for B-A transport). HBSS pH 7.4 was added to the receiver wells and run for 2 h at 37 °C without shaking. Collection of samples was done at the start (8 µL from the donor only) and at the end (8 µL and 80 µL from donor and receiver, respectively). Samples from the donor wells required dilution (in HBSS pH 7.4) and added to acetonitrile containing internal standard for LC-MS/MS analysis. The peak area ratios of the analyte/internal standard were utilised for calculating the *P*_app_ values. The ER is the ratio of B-A P_app_/A-B P_app._

### Liquid chromatography with electrospray mass spectrometric detection

Chromatography was performed on a Waters Acquity UPLC pump (Waters, Milford, MA, USA) on a C-18 Kinetex column (50 ×2.1 mm, 2.6 µm) kept at a temperature of 50 °C. The mobile phase consisted of A) 0.1% formic acid in water and B) 0.1% formic acid in methanol (100% v/v). The gradient elution program at a flow rate of 0.6 mL/min began with 95% A for 0.3 min, a decrease to 5% in 1.0 min, held at 5% for 1.0 min, back to 95% in 0.01 min and held for 0.5 min to give a total run time of 2.8 min. Mass spectrometric detection was done in positive mode on a Waters TQ-XS with data acquisition performed using MassLynx V4.1. The gas flow rates were 600 L/h for desolvation and 50 L/h for cone. The desolvation and source temperatures were set at 350 and 120 °C, respectively. The capillary voltage was 3.27 kV and the analytes were monitored by multiple reaction monitoring.

Quantitation of savolitinib metabolites M2 and M4 was done using authentic standards synthesized at AstraZeneca, purity: 97% (M2), 100% (M4), for synthesis, see patents (Hutchison Medipharma Limited World Intellectual Property Organization, WO2011079804 A1 and ASTRAZENECA - WO2020/53198, 2020, A1). For 1´-hydroxy midazolam and the glucuronide of 1´-hydroxymidazolam, the expected transitions were monitored by response ratios (metabolite peak area/peak area of internal standard).

### Incubations for metabolite identification studies

The reaction mixtures for HLM with NADH/NADPH and human hepatocytes were performed as outlined but with a final concentration of AZ15 (and savolitinib for hepatocytes only) of 5 µM in a final volume of 250 µL. After a pre-incubation at 37 °C for 5 min, the reaction was started by adding the test compound. After 60 min, the reaction was stopped by adding an equal volume of 100 µL ice-cold acetonitrile (without internal standard). After centrifugation at 3000 × *g* for 10 min, 200 µL of the supernatant was mixed with 400 µL of water before analysis for metabolites.

### Metabolite identification by liquid chromatography mass spectrometry

Accurate mass structural characterisation was carried out on a Thermo Scientific Orbitrap Fusion mass spectrometer connected to a Waters Acquity UHPLC system containing a photodiode array detector, column manager and binary solvent manager. The separations were carried out as previously described. Briefly, chromatography was performed on a Waters BEH C18 (100 × 2.1 mm, 1.7 µm) with the column temperature kept at 60 °C.

### Live cell fluorescence lifetime imaging

To prepare the cells, 3 vials of cryopreserved human hepatocytes were thawed rapidly in a 37 °C water bath and transferred into pre-warmed Leibovitz media by pouring followed by purification by centrifugation and resuspension by gentle rocking. Trypan blue exclusion indicated cell viabilities of 80–90%. Cell suspensions were transferred to 4 cm tissue culture dishes (Falcon, Fisher Scientific) for imaging^61,74^.

### Statistics and reproducibility

The graphs were plotted in GraphPad Prism Version 8.4.3 (GraphPad Software Inc., La Jolla, CA, USA). Statistical analysis was also performed in GraphPad Prism, and statistical significance was assessed by the Brown-Forsythe and Welch ANOVA followed by Dunnett’s T3 test for multiple comparisons and a *P* < 0.05 was considered significant. For comparisons of two groups, an unpaired two-tailed *T*-test with Welch’s correction was used and a *P* < 0.05 was considered significant. Pooled biological matrices were used (details provided in methods sections): hepatocytes (two different lots with *n* = 10 and one lot with 20 donors; liver microsomes, two lots with *n* = 150 donors, one lot with *n* = 50) and liver S9 (pool of 50 donors). For human hepatocytes, each replicate was from an independently resuscitated vial of hepatocytes incubated separately with duplicate technical replicates. All other matrices were also taken from fresh vials incubated separately with duplicate technical replicates. For Fig. 4, the large number of compounds restricted the replicates to one. However, as the data from the other figures shows very good reproducibility, data from the single determination for each compound should give an accurate comparison of the rates of metabolism with the two different cofactors in HLM relative to HH. Details are provided in the legends of each figure.

## Supplementary information

Supplementary information

Description of Additional Supplementary Files

Supplementary Data 1

Reporting summary

## Data Availability

Data supporting the findings of this study are available within the paper and its supplementary information. The source data is provided as supplementary data 1. Any other data are available from the corresponding author upon request.
