# Supplementary material for: A mismatch in enzyme-redox partnerships underlies divergent cytochrome P450 activities between human hepatocytes and microsomes
Source: Commun Biol. 2025 Nov 6;8:1539. doi: 10.1038/s42003-025-08903-1 (PMC12592420; doi:10.1038/s42003-025-08903-1)
Supplement: Supplementary file 1 — Supplementary information [file 42003_2025_8903_MOESM1_ESM.pdf]

Supplementary Figure 1: Increasing concentration of human cytosolic protein reduces the ratio of savolitinib metabolites (M4:M2) formed by human liver microsomes

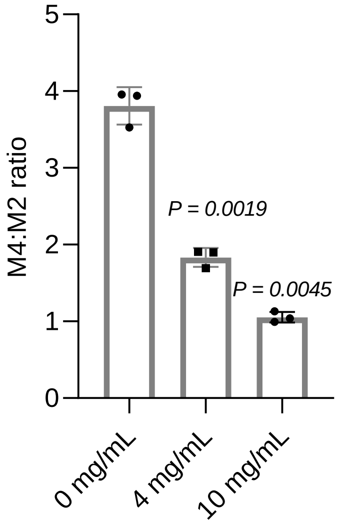

The effect of increasing concentrations of human cytosolic protein on the ratio of savolitinib metabolites (M4:M2) formed by human liver microsomes (1 mg/mL) fortified with NADPH at 37°C for 1 hr. The data represent an average ± SD of three independent duplicate measurements. Statistical significance was assessed by the Brown-Forsythe and Welch ANOVA followed by Dunnett's T3 multiple comparison of the control (0 mg/mL) to the other groups (4 and 10 mg/mL).

Supplementary Table 1: Intrinsic clearance (CL_{int}) of savolitinib by a panel recombinant human cytochrome P450 enzymes

Enzyme	CL_{int} (µL/min/pmol CYP)	% contribution [a]
CYP1A2[b]	0.0386	1.6
CYP2A6[c]	0.0148	0.2
CYP2B6	NC	NC
CYP2C8	NC	NC
CYP2C9	NC	NC
CYP2C19	0.134 ± 0.0365	1.9
CYP2D6	0.309 ± 0.0010	1.8
CYP2E1	NC	NC
CYP3A4	0.489 ± 0.0278	83
CYP3A5[b]	0.0939	12

CL_{int} values are average of three independent determinations.

NC: not calculated due to insignificant turnover

[a] Percentage contribution calculated using inter-system extrapolation factors[1, 2].

[b] only two experiments produced measurable CL_{int} values

[c] only one experiment produced measurable CL_{int} value

Supplementary Table 2: Efflux ratios of midazolam (0.1 µM) in MDCKII-MDR1-BCRP and NIH MDCKI-MDR1 cell lines

MDCKII-MDR1-BCRP	NIH MDCKI-MDR1
0.50	1.24
0.52	1.26

Showing results determined from individual wells.

Supplementary Table 3: Compound physicochemical properties

Compound	Mw	LogD pH 7.4	Ion Class	Caco-2 P_{app} (10^{-6} cm/s)
Chlorpromazine	318.9	3.35	Base	nd
Caffeine	194.2	<-0.0108	Neutral	81.5
Oxaprozin	293.3	1.2	Acid	nd
Propanolol	259.4	1.28	Base	45.4
Nifedipine	346.3	3.15	Neutral	78.3
Gefitinib	446.9	3.77	Base	nd
Verapamil	454.6	2.6	Base	20.9
Amodiaquine	355.9	2.9	Base	15.098
Diazepam	284.8	2.7	Neutral	34.2
Benzydamine	309.4	2.25	Base	27.422
Imipramine	280.4	2.5	Base	33.4
Phenacetin	179.2	1.48	Neutral	76.1
Warfarin	308.3	0.902	Neutral	60.7
Furosemide	330.8	<-0.596	Acid	1.42
Metoprolol	267.4	<-0.190	Base	19.52
Diflunisal	250.2	0.574	Acid	129
Adavosertib	500.6	2.37	Base	12.7
Zimlovisertib	361.4	1.98	Neutral	31.7
AZ12	408.5	2.4	Base	16.8
AZ13	393.5	1.85	Base	20.2
AZ14	457.5	2	Base	22.263
AZ15	475.5	2.3	Base	31.661
AZ16	489.6	2.6	Base	33.635
AZ17	445.5	2.3	Base	11.564
AZ18	421.5	2.32	Neutral	44.6
AZ19	459.6	2.94	Base	7.22
AZ20	370.5	3.1	Neutral	61.097
Revumenib	630.8	1.41	Base	1.651
AZ22	559.7	1.1	Base	0.439
AZ23	412.4	3.08	Neutral	66.417
AZ24	559.7	1	Base	0.336
AZ25	573.7	1.1	Base	0.589
AZ26	611.8	1.9	Base	nd
AZ27	585.7	2.1	Base	nd
AZ28	630.8	0.75	Base	<0.501
AZ29	398.5	2.32	Neutral	73.381
AZ30	669.8	2.95	Base	10.138
AZ31	559.7	1.9	Base	2.563
AZ32	430.4	3.6	Neutral	72.581

AZ33	616.8	1.1	Base	0.978
AZ34	587.7	1.7	Base	0.785
AZ35	570.6	2.9	Base	nd
AZ36	604.6	3.4	Base	7.846
AZ37	558.7	0.65	Base	0.351
AZ38	554.7	1.1	Base	0.623
Bleximenib	599.8	0.74	Base	0.575
AZ40	557.7	1.3	Base	1.263
AZ41	464.9	1.66	Base	20.939
Diclofenac	296.2	1.07	Acid	118
Prazosin	383.4	1.88	Neutral	34.587
Ketanserin	395.4	2.9	Base	32.676
Lorazepam	321.2	2.38	Neutral	67.2
Troglitazone	441.6	4.25	Neutral	nd
Midazolam	325.8	3.4	Neutral	47.5
Bufuralol	261.4	1.7	Base	33.8
Carvedilol	406.5	3.27	Base	nd
Naloxone	327.4	1.23	Base	11.9
Ritonavir	721.0	4.05	Neutral	nd
Sildenafil	474.6	2.74	Base	66
Trovafloxacin	416.4	0.47	Zwitterion	nd
Cefoperazone	645.7	-2.52	Acid	nd
AZ51	503.7	2.35	Base	nd
AZ52	362.4	1.3	Base	nd
Savolitinib	345.4	1.81	Neutral	53.7

nd: not determined

LogD pH 7.4 and Caco2 P_{app} values were from at least one determination

Supplementary Methods

Effect of cytosolic protein on formation of savolitinib metabolites M4:M2 ratios. The reaction mixture consisted of human liver microsomes (pool of 150 donors, batch QQY from BioIVT, final concentration 1 mg/mL), potassium phosphate buffer pH 7.4 (final concentration 0.1 M), NADPH (1 mM), savolitinib (1 μM). Human liver cytosol (Corning, 452115, suspended in 50 mM Tris-Cl pH 7.5, 150 mM KCl, 2 mM EDTA) was added to give final concentrations of 4 and 10 mg/mL were compared to the control (with 50 mM Tris_Cl pH 7.5, 150 mM KCL, 2 mM EDTA in place of the cytosolic protein). After a preincubation of 5 min at 37°C, the reaction was started by adding savolitinib. An aliquot (25 μL) was taken after 1 hr and added to ice-cold acetonitrile containing internal standard (100 μL). After centrifugation at 3000 x g for 10 min, the supernatant was transferred to a clean 96-well plate and diluted 6-fold with water. After mixing, 3 μL was injected into a mass spectrometer. Liquid chromatography with mass spectrometric detection. Chromatography was performed on a waters Acquity UPLC pump (Waters, Milford, MA, USA) on a C-18 Kinetex column (50 x 2.1 mm, 2.6 μm) kept at a temperature of 50°C. The mobile phase consisted of A) 0.1 % formic acid in water and B) 0.1 % formic acid in methanol (100 % v/v). The gradient elution program at a flow rate of 0.6 mL/min began with 95% A for 0.3 min, a decrease to 5% in 1.0 min, held at 5 % for 1.0 min, back to 95 % in 0.01 min and held for 0.5 min to give a total run time of 2.8 minutes. Mass spectrometric detection was done in positive mode on a Waters TQ-XS with data acquisition performed using MassLynx V4.1. The gas flow rates were 600 L/h for desolvation and 50 L/h for cone. The desolvation and source temperatures were set at 350 and 120°C, respectively. The capillary voltage was 3.27kV and the analytes were monitored by multiple reaction monitoring.

Quantitation of savolitinib metabolites M2 and M4 was done using authentic standards synthesized at AstraZeneca.

Screen to identify CYPs metabolising savolitinib. The reaction mixture consisted of recombinant enzyme from BioIVT, formerly Cypex (final 100 pmol/mL) from bactosomes prepared from Escherichia. coli, potassium phosphate buffer pH 7.4 (final concentration 0.1M), NADPH (1 mM) and test compound (1 μM). The reaction was started by addition of test compound after a pre-incubation of 5 min at 37°C. Aliquots (25 μL) were taken at 0, 5, 10, 15 and 25 minutes and added to ice-cold acetonitrile containing internal standard (100 μL). After centrifugation at 3000 x g for 10 min, the supernatant was transferred to a clean 96-well plate and diluted 6-fold with water. After mixing, 3 μL was injected into a mass spectrometer. The panel of enzymes included CYPs 1A2 (CYP001, Lot C1A2R010C), 2A6 (CYP011, Lot C2A6R008D), 2B6 (CYP020, Lot C2B6R046), 2C8 (CYP017, Lot C2C8R005), 2C9 (CYP019, Lot C2C9H028/A), 2C19 (CYP008, Lot C2C19R016D), 2D6 (CYP007, Lot C2D6R030), 2E1 (CYP009, Lot C2E1R017), 3A4 (CYP002, Lot C3A4R046B) and 3A5 (CYP046, Lot C3A5R004). The clearance of savolitinib was calculated from the peak area determined by LC-MS/MS. The average intrinsic clearance was used from three independent determinations. For each CYP, the percentage contribution to the total metabolism of savolitinib was calculated by using the intersystem extrapolation factors and abundance of the CYP or each CYP, the percentage contribution to the total metabolism of savolitinib was calculated as described[1, 2].

Determination of efflux ratio in MDCKII-MDR1-BCRP and NIH MDCKI-MDR1 cell lines. The MDCKII-MDR1-BCRP[3] and NIH MDCKI-MDR1[4] cell lines were used for assessment of efflux. After confirmation of cell monolayer integrity, compounds were dissolved in Hank's balanced salt solution (HBSS) pH 7.4 to achieve appropriate concentrations (final 0.1 μM). Transport assays were done by adding the working solution to the apical chamber (to assess A-B transport) and to the basolateral chamber (for B-A transport). HBSS pH 7.4 was added to the receiver wells and run for 2 h at 37°C without

shaking. Collection of samples was done at the start (8 µL from the donor only) and at the end (8µL and 80 µL from donor and receiver, respectively). Samples from the donor wells required dilution (in HBSS pH 7.4) and added to acetonitrile containing internal standard for LC-MS/MS analysis. The peak area ratios of the analyte/internal standard were utilised for calculating the Papp values. The ER is the ratio of B-A Papp/A-B Papp.

Determination of intrinsic permeability. The intrinsic permeability of savolitinib was measured in Caco-2 cells in which efflux transporters were inhibited. Compound (10 µM) was added to the apical side in the presence of the P-glycoprotein inhibitor quinidine (50 µM), MRP2 inhibitor benzbromarone (30 µM) and breast cancer resistance protein inhibitor sulfasalazine (20 µM) and incubated at 37°C. Aliquots were taken at 0 and 2 h and added to acetonitrile containing internal standard. After centrifugation, the supernatant was analysed by LC-MS/MS. The apparent permeability (Papp) was calculated as follows:

Papp=VA/(Area x time) x [drug]acceptor/([drug]initial,donor)

Where VA = volume (mL), Area = surface area of membrane and time (incubation time in second) [5].

Determination of LogD pH 7.4. The octanol/phosphate buffer (pH 7.4) was used to determine the LogD[4]. Briefly, equal volumes of octanol (saturated with phosphate buffer pH 7.4) and phosphate buffer pH 7.4 (saturated with octanol) were added to 96 deep-well plates. After adding compound (dissolved in DMSO, final 1% v/v), the plates were sealed and vortex-mixed for 30 min. Equilibration for 3 h on a horizontal shaker was followed by centrifugation (3220 x g for 30 min). Samples from both phases were analysed by LC-MS/MS and the LogD was calculated as log of [compound] in octanol/[compound] in phosphate buffer.

Supplementary References

1. Chen Y, Liu L, Nguyen K, Fretland AJ. Utility of intersystem extrapolation factors in early reaction phenotyping and the quantitative extrapolation of human liver microsomal intrinsic clearance using recombinant cytochromes P450. *Drug Metab Dispos* **39**, 373-382 (2011).

2. Proctor NJ, Tucker GT, Rostami-Hodjegan A. Predicting drug clearance from recombinantly expressed CYPs: intersystem extrapolation factors. *Xenobiotica* **34**, 151-178 (2004).

3. Colclough N*, et al.* Utilizing a dual human transporter MDCKII-MDR1-BCRP cell line to assess efflux at the blood brain barrier. *Drug Metab Dispos* **52**, 95-105 (2024).

4. Feng B*, et al.* Validation of human MDR1-MDCK and BCRP-MDCK cell lines to improve the prediction of brain penetration. *J Pharm Sci* **108**, 2476-2483 (2019).

5. Bapiro TE*, et al.* The disconnect in intrinsic clearance determined in human hepatocytes and liver microsomes results from divergent cytochrome P450 activities. *Drug Metab Dispos* **51**, 892-901 (2023).
